# A New Frontier: The Convergence of Nanotechnology, Brain Machine Interfaces, and Artificial Intelligence

**DOI:** 10.3389/fnins.2018.00843

**Published:** 2018-11-16

**Authors:** Gabriel A. Silva

**Affiliations:** Departments of Bioengineering and Neurosciences, Center for Engineered Natural Intelligence, University of California San Diego, La Jolla, CA, United States

**Keywords:** nanotechnology, neuroscience, machine learning, artificial intelligence (AI), brain machine interface (BMI), brain computer interface, computational neuroscience

## Abstract

A confluence of technological capabilities is creating an opportunity for machine learning and artificial intelligence (AI) to enable “smart” nanoengineered brain machine interfaces (BMI). This new generation of technologies will be able to communicate with the brain in ways that support contextual learning and adaptation to changing functional requirements. This applies to both invasive technologies aimed at restoring neurological function, as in the case of neural prosthesis, as well as non-invasive technologies enabled by signals such as electroencephalograph (EEG). Advances in computation, hardware, and algorithms that learn and adapt in a contextually dependent way will be able to leverage the capabilities that nanoengineering offers the design and functionality of BMI. We explore the enabling capabilities that these devices may exhibit, why they matter, and the state of the technologies necessary to build them. We also discuss a number of open technical challenges and problems that will need to be solved in order to achieve this.

## Introduction

A confluence of technological capabilities is creating an opportunity for machine learning and artificial intelligence (AI) to enable “smart” nanoengineered brain machine interfaces (BMI). The goal is for this new generation of technologies to be able to communicate with the brain in ways that support contextual learning and adaptation to changing functional requirements. This applies to both invasive technologies aimed at restoring neurological function, as in the case of neural prosthesis, as well as non-invasive technologies enabled by signals such as electroencephalograph (EEG). Advances in computation, hardware, and algorithms that learn and adapt in a contextually dependent way will be able to leverage the capabilities that nanoengineering offers the design and functionality of BMI. Eventually, these technologies will be able to carry out learning and adaptation in (near) real time, as external shifting demands from the environment and physiology require them. Ultimately, the goal is to produce personalized individual user experiences for applications such as gaming, and to allow the device to learn and adapt to changing disease requirements in clinical scenarios. In this commentary we explore the enabling capabilities that these devices may exhibit, why they matter, and the state of the technologies necessary to build them. We also discuss a number of open technical challenges and problems that will need to be solved in order to achieve this.

## The Opportunity for “Smart” Brain Machine and Brain Computer Interfaces

Brain machine and brain computer interfaces (we use these terms interchangeably here) represent technologies designed to communicate with the central nervous system: the brain, spinal cord, and neural sensory retina. Clinically, depending on the design and intent of the technology, the goal can be to record and interpret neural signals in order to execute an intended neural command through an external device, or to achieve neural stimulation, often to restore neural function following disease or trauma, or both (Adewole et al., 2016; Choi et al., 2017; Slutzky and Flint, 2017; Rezeika et al., 2018). Some devices make use of feedback in an attempt to optimize performance, whether physiological or via patient specific intent and instructions (Widge et al., 2018). There is also a growing list of non-invasive brain machine interface technologies not meant for clinical use, primarily driven by innovative startup companies. These technologies are intended to augment the user experience and control interface for gaming and augmented (AR) and virtual reality (VR) applications. Although of course very different than technologies aimed at treating and restoring clinical function and quality of life to patients, this is a market that should not be ignored. Not the least of which because it could provide leveraging resources to the benefit of clinically related research. For example, advances in our understanding of the relevant neurophysiology, cognitive neuroscience, mathematical and engineering aspects of signal processing, and hardware, can significantly impact both the gaming industry as well as clinical devices and neural prosthesis. The brain machine interface market is projected to reach $1.46B by 2020, with a compound annual growth rate (CAGR) of 11.5% between 2014 to 2020 by one estimate (Allied Market Research, 2015), and a comparable $1.72B in 2022, with a predicted CAGR of 11.5% between 2012 and 2022 by another estimate (Grand View Research, 2018). Much of this projected growth will be due to non-invasive technologies, with the gaming industry as a market driver roughly on par with healthcare applications.

As significant as these numbers are, these projections primarily reflect enabling technologies for interfacing between neural control and sensory experiences with machines. They do not reflect opportunities that go beyond what is currently possible with the existing state of the art. BMI that can learn and adapt reflect the cutting edge of what is technologically possible, due to a confluence of BMI technologies, in particular nanotechnologies, machine learning and AI, alongside a continued increasing understanding of the relevant neuroscience. AI can provide opportunities to create “smart” BMI that contextually learn and adapt to changing functional requirements and demands. This has the potential to produce personalized individual experiences in gaming and AR/VR, and allow for the changing requirements associated with patient specific disease progression and evolution in clinical applications. This latter point cannot be overestimated, because not only would it accommodate the differing clinical demands of different neurological disorders, it would allow for patient specific adaptation of BMI functionality to the needs of different patients. And it would allow the technology to continue to adapt as disease progression evolves in individuals over time. One of the significant limitations of current state of the art BMI and neural prosthesis is the assumption of one size fits all. In in other words, the assumption that a technology operating under a specific set or range of functionality will properly treat all patients. While we are not aware (yet) of a device or technology that reflects the actualized integration of machine learning and nanotechnology applied to BMI, we argue that the potential and impact of doing make the subject worth exploring. Each on their own, machine learning and nanotechnology, are already being used in the design and function of BMI and neural prosthesis in a number of ways that align with the vision we propose here.

## Integration of Bmi With Machine Learning

What advantages does machine learning and artificial Intelligence (AI) offer BMI? What exactly are machine learning algorithms learning, and how can they use that information to adapt in a meaningful way? What these algorithms can learn is information provided by feedback and telemetry from the hardware. This could be information about the current state of the output settings of the device, or any kind of external information measured by sensors in the BMI. For example, physiological measurements in response to stimulation, feedback from other algorithms external to the BMI-machine learning system, such as haptic or computer vision feedback, or the internal parameter settings of current stimulation or recording protocols to the device. In the case of internal parameters the algorithms have constant access to variables such as pulse durations and amplitudes, stimulation frequencies, energy consumption by the device, stimulation or recording densities, electrical properties of the neural tissues it is interfacing with (resistances, impedances), and continuous or near continuous levels of biochemical factors such as neurotransmitters or other metabolites. Of course, none of these are mutually exclusive, with multiple types and streams of information possibly being provided to the algorithms in parallel, albeit at likely different sampling resolutions. With this information, machine learning algorithms could then identify subtle and non-trivial patterns and phenomena in the data, ideally in (near) real time, in order to produce desired functional outcomes from the BMI that change dynamically as external (e.g., clinical or functional) requirements demand them. This would necessitate the development and training of machine learning models and algorithms offline as part of the design of the BMI system. The algorithms would need to learn a wide enough range of the parameter spaces in order to appropriately identify patterns in the data they encounter when online. Subsequent algorithms can then autonomously make decisions about how to use that data. This step does not necessarily have to be part of the brain machine interface system itself, and could be executed with algorithms computing in the cloud, if sufficient bandwidth was available. Or even offline following periodic data downloads, for example. Clearly though, on board decision algorithms that operate in real time with the machine learning algorithms that are identifying patterns in the data would be ideal. This would alleviate issues of data transfer delays, and bandwidth insufficiency. This could also allow for the need to store less data on the device, which could be limited due to physical constrains. Data would only have to be stored long enough for the system to make an autonomous decision, essentially as a moving window that matches the processing capabilities of the algorithms. Of course, it may still be valuable or necessary to store some data or types of data for offline analyses even though they may not be needed for the BMI system to make a decision. For example, in order to understand offline after the fact why the algorithms made the decisions they made and the clinical outcomes of those decisions. With this process complete we close the loop: information is provided to the machine learning algorithms, followed by learning, pattern identification, and subsequent executable autonomous decisions that in turn dynamically change the output of the brain machine interface and how it interacts with the external environment it is interfacing with. This could be the brain itself in the case of a neural prosthesis intended to restore clinical function, or software in a non-invasive BMI that is interfacing that is part of an AR or VR system.

While still early days, a number of research groups have recognized this potential, and are beginning to explore how machine learning could inform and integrate neural stimulation and feedback. Nurse and colleagues (Nurse et al., 2015) have developed a generalized approach that takes advantage of a stochastic machine learning method to classify motor related signals specifically for BMI applications. Importantly, their classifier does not need to rely on the use of extensive *a priori* data to train the BMI. Their algorithms outperformed other methods on the Berlin BMI IV 2008 dataset, and demonstrated high levels of classification accuracy when tested on datasets derived from EEG signals. In another recent study, Ortega et al. (2018) explored different data pre-processing strategies and convolution neural network architectures for classification tasks derived from EEG signals. Interestingly, they found that a rather straightforward network architecture, when combined with a pre-processing step that analyzed spectral power preserving features of the electrode arrangements, was sufficient to handle the analysis of the data. Their network consisted of a single convolution layer, one connection layer, and single linear regression classifier layer. Their approach allowed them to carry out co-adaptive training on the data to achieve on-line classification. A different study had explored a similar approach. Lawhern et al. (2016) carried have also explored a similar approach.

As discussed above, one of the biggest advantages machine learning may confer on BMI is the ability to achieve real-time or near-real-time modulation of output or stimulation parameters in response to active real-time feedback from physiological signals, the environment, or other internal cues from the system itself, such as possibly the output from other internal algorithms that have processed some amount of data. Most BMI’s have a decoder component whose job it is to decode and make sense of neural signals in order to produce executable or actionable outputs. This typically necessitates extensive supervised training in order to optimize the interpretation of recorded neural signals before the decoder can properly correlate observed signals with desired outputs and commands. This training has traditionally required supervised feedback with a human in the loop, typically a technician or clinician often with input from the patient her/himself, thus making the process highly inefficient and intermittent. Training and subsequent adjustments can only occur periodically and are typically time consuming. Furthermore, the mapping from neural signals to actionable outputs is limited to the training data the system is exposed to during the training. This then highly limits the ability of the BMI to respond to variable real world scenarios it may encounter when in use, thus severely limiting its functionality to the patient when such conditions arise. Early work relied on feedback from external sensory references to compute an error between the output of the system and desired supervised target. These included visual and auditory signals (Wessberg et al., 2000; Lebedev et al., 2005), mechanotransduction (Nicolelis and Chapin, 2002; O’Doherty et al., 2011), and direct cortical sensory stimulation (6 Bach-y-Rita and Kercel, 2003). But these approaches are severely limited due to their need for continuous information from an external reference target to adjust the mapping to the output of the BMI. More recent work has addressed some of these limitations by adapting output parameters to unsupervised learning methods such as Bayesian statistical methods and reinforcement learning that do not rely on an external reference (Vidaurre et al., 2011; Orsborn et al., 2012, 2014; Bryan et al., 2013; Huang and Rao, 2013; Bauer and Gharabaghi, 2015). Although in most cases they still require significant training periods. More recent studies have begun to investigate the use of endogenous neural signals directly as the training source in iterative closed feedback loops with the BMI that can respond and adapt in a much more direct way (Suminski et al., 2010; Carmena, 2013). For example, Prassad and colleagues are developing an approach they call Actor-Critic reinforcement learning that does not need to rely on a supervised error signal (Pohlmeyer et al., 2014; Prins et al., 2014, 2017).

In general, the BMI field in general and neural prosthesis field in particular are still exploring machine learning. One of the challenges is that key state of the art methods, such as deep learning, that have had huge successes in other applications may not be the best approach for the constraints imposed by the needs of BMI (Vidaurre et al., 2015). In a recent paper, Panuccio et al. (2018) do an excellent job summarizing the current state and challenges of neural engineering aimed at restoring neural function, including proposing a number of similar requirements discussed in the current paper, that emerging algorithms and machine learning will need to address in order to build a true adaptive BMI.

An important consideration that such machine learning approaches offer that other methods cannot is the opportunity to develop BMI that adapt to the scaling requirements, both spatial and temporal, necessitated to achieve targeted functional outcomes. In the context of neural stimulation, the optimal density of stimulation required to produce a target response in neuronal populations being stimulated is a complex consideration, and may not always be the highest stimulation density achievable by the device (Shepherd et al., 2013; Patil and Thakor, 2016). What the right stimulation density should be can be a complex question to answer, and often depends on specific physiological and pathophysiological considerations. In many situations we still do not fully understand what the right stimulation density should be and why. Furthermore, the optimal stimulation density is likely to vary from individual to individual even in the same disorder, and within an individual patient the disease can greatly evolve over time as the physiology changes and the body responds and adapts to altered conditions. This could be a function of age or exogenous perturbations such as a response to other treatments, diet, and the psychological state of the patient. Another consideration is that hardware or other algorithms that need to make use of recorded or measured neural data in order to interact with the brain could have different scaling requirements in the data. This would be dependent on what the external query is and how the neural data needs to be used. It reflects the technical capabilities and limitations of the external technologies requesting the data. Under sampling could lead to poor user interactions, for example, a frustrating or confusing AR/VR experience, or the inability of a disabled patient to communicate in a timely or accurate manner. Oversampling would waste computational resources and time. In a research setting, data scaling issues could affect the empirically determined accuracy of a computational model, or how a hypothesis is tested and interpreted. Clinically, it could impact treatment or other clinical decisions. Changing temporal and spatial scaling requirements demanded by exogenous considerations to the BMI present situationally unique challenges that the existing state of the art is not yet able to address in a substantive way. The integration of machine learning and AI with nanoengineered BMI offers the opportunity for these technologies to learn, adapt, and respond to their environments in order to address functionally challenging considerations such as dynamic scaling demands.

## Beyond the Current State of the Art: Machine Learning Enabled Nanoengineered Bmi

In recent years there has been an explosion of work focused on the development and use of nanotechnologies aimed at interacting and interfacing with the brain and central nervous system generally (Silva, 2006, 2007a,b, 2008, 2010; Kotov et al., 2009; De Vittorio et al., 2014; Saxena et al., 2015; Badry and Mattar, 2017; Scaini and Ballerini, 2017; Rosenthal, 2018), and in the context of BMI and neural prosthesis in particular (Webster et al., 2003; Lovat et al., 2005; Fabbro et al., 2012; Nicolas-Alonso and Gomez-Gil, 2012; Seo et al., 2013; Avants et al., 2016; Ha et al., 2016; Scaini and Ballerini, 2017). Considerable recent effort has focused on nanoscale neurotechnologies aimed at recording from and stimulating from the brain at high densities. This has to a significant degree been motivated by federal research efforts in both the United States and the European Union through the Brain Initiative^1^ and Human Brain Project,^2^ respectively. We do not attempt to review this extensive literature here, but refer the reader to the references and published literature more broadly.

While the confluence of machine learning and nanoengineered BMI and neural prosthesis has not yet occurred, machine learning is playing an increasing role in other aspects of nanotechnology and related molecular-scale research (for example, see the review by Sacha and Varona, 2013). In one example, Albrecht et al. (2017) have written a tutorial for using deep learning convolution neural networks for analyzing and mining single molecule data from DNA sequencing experiments. Ju et al. (2017) recently showed they could use an atomic version of Green’s function and Bayesian optimization to optimize the interfacial thermal conductance of Si-Si and Si-Ge nanostructures (Ju et al., 2017). Their method was able to identify optimal structures within a library of over 60,000 candidate structures. And in another striking recent study, Lin et al. (2018) were able to implement a deep learning architecture on an all-optical 3d printed Diffractive Deep Neural Network (D2NN) that were designed and optimized by deep learning. These researchers were able to carry out classification and other imaging tasks without the need or use of any power, except for the input light into their system. The opportunity for BMI and neural prosthesis lies in the ability of machine learning to “learn” (i.e., identify and classify patterns) in highly complex physical and chemical data derived from devices that have been engineered at the nanoscale in order to inform and optimize the design and functional outputs of the devices.

As already alluded to, however, an important consideration in this quest is the realization that existing machine learning and AI algorithms may not be optimal for such needs. Thus, there is a possible opportunity and need for the development of purposely developed machine learning algorithms specifically designed to take advantage of and control nanoengineered BMI devices. Current machine learning methods, in particular deep artificial neural networks (ANN’s), are incredibly powerful and continue to show some spectacular progress. What is probably the most surprising is that at its most fundamental, the underlying learning rules responsible for the existing state of the art and success of ANN’s are essentially all variants of gradient descent statistical learning methods. But like any method, there are theoretical and practical limitations. The data they operate on must therefore be able to accommodate these constraints. In particular, existing algorithms are dependent on exposure to enormous data sets to train them properly so they can learn (a form of model bias). They can only find associations and patterns in the data that already exist (model bias again). There is always a danger of over generalizing from a limited training set (model over fitting). A such, they display an almost complete lack of robustness and ability to adapt beyond the training sets they are exposed to. New data may not achieve further learning (model saturation). And because of these considerations these methods will miss outliers (data sparseness problem). Finally, they require large computational resources and the consumption of huge amounts of energy to properly identify learned patterns. These methods are limited by a set of fundamental engineering challenges inherent to statistical learning. Yet, even with these constraints in mind, and ignoring all the hype currently surrounding machine learning and AI, it is difficult not to be impressed by the accomplishments these methods are achieving. If the data and resources are appropriate to the task being presented to the algorithms, these methods can work remarkably well and it is likely impractical (and even unnecessary) to attempt to develop new methods to supersede them; at least for the foreseeable future. In some cases it is certainly plausible that the machine learning needs of nanoengineered BMI’s could be amenable to the current state of the art (see references and discussion above). BMI can generate significant amounts of data, and the range of operating conditions of physiological signals, stimulation parameters, and recording densities specific to given functional tasks are sufficiently well understood, at least from the perspective of defining the extremes of those conditions. Thus, sufficient data over known and practical physiological operating ranges could allow existing machine learning to learn sufficiently in order to guide decision algorithms for adapting the interactions of the BMI with their targets. This is particularly true of nanoengineered brain machine interface’s, whereby the degree of synthesis control over the material or device, and spatial and temporal stimulation resolutions and recording densities, can be engineered at the nanoscale. Conceivably, the quality and amount of information nanoengineered BMI could produce, along with the degree of functional control nanoscale engineering provides, are particularly well suited to take advantage of the state of the art in machine learning and AI in order to achieve smart integrated BMI.

At the same time, however, it is worth asking if machine learning and AI architectures designed to learn differently than existing algorithms could provide a degree of functionality and integration with BMI’s that does not yet exist. In particular, machine learning methods that mathematically model and abstract specific neurobiological properties of interest. Empirical (i.e., data driven) statistical learning AI works well on problems where bias, sparseness, and saturation are not (or not yet) an issue that limit its learning. But it is precisely learning beyond these constraints that the biological brain excels at. In particular, the ability of the brain to adapt and extrapolate beyond data presented to it, and it is incredible computational and physical robustness to perturbations. These properties go beyond the current stage of the art in machine learning, but could be critical to the sophisticated integration of BMI with the brain. The biological brain represents, learns, and manipulates information very differently than the way existing artificial neural networks, machine learning, and statistical methods “learn” to find patterns in data. The brain primarily learns by analogy and by abstracting beyond the immediate training sets presented to it. It is capable of robustly adapting to different situations and contexts it may not have previously encountered with an incredible degree of plasticity. The computational flexibility, adaptation, and robustness of the brain exceed any existing machine. One extreme example of the human brain’s incredible robustness and ability to adapt is evident in a neurological condition called Rasmussen’s encephalitis, a rare pediatric chronic inflammatory neurological disorder that typically affects one hemisphere. It is typically characterized by severe and frequent seizures that result in loss of motor function, loss of speech, hemiparesis, encephalitis, and cognitive decline (Freeman, 2005; Varadkar et al., 2014; Venkatesan and Benavides, 2015). Most patients become refractory (stop responding) to medical treatment. In many cases the only effective treatment for seizures is hemispherectomy, whereby portions or the entire affected cortical hemisphere are surgically removed and the corpus callosum cut from the unaffected hemisphere. The corpus callosum is the high speed “ribbon cable” that connects our two sides of the brain. Yet, to varying degrees, the remaining side of the cortex in these patients is able to take up the functions of the excised cortical tissue to a remarkable extent. In many cases these patients are able to function cognitively and physically almost normally considering how much of their brains are removed. (Contrast that with what would happen if you remove even a handful of the transistors or circuits in a computer.) All of this is even more impressive given the computational and energy efficiency with which the brain achieves this - using about 20 watts of power, barely enough to power a dim light bulb, in about 3 lbs of “wetware” that occupies a volume equivalent to a 2 liter bottle of soda.

One final comment worth emphasizing is that although the biological brain exhibits computational properties and an ability to learn that we want to understand and leverage, this does not necessarily mean that we have to reverse engineer the brain to the point that we are modeling or emulating every aspect of how the biology itself implements the brain’s internal algorithms. One approach is to abstract away the biological details and capture the core algorithms, i.e., rules, that underlie the property or system being studied in the brain that we want to build into the BMI. The end result are mathematical models that are independent of the underlying biological details, but which capture the functional mechanisms at an appropriate scale of abstraction in order to arrive at algorithmic descriptions that emulate those properties. Admittedly, where that line of abstraction is drawn can be more an art than a science.

## Challenges and Open Problems

In this final section we briefly introduce some of the challenges and open problems associated with actually executing the vision discussed above. We do not elaborate in this paper, but leave them open for further discussion and dialog.

First, most (all) of the recent efforts in the development of neurotechnologies aimed at high density recording or stimulation have focused on the physics, chemistry, and engineering of the core nanotechnologies themselves. This is understandable because the fundamental technologies necessary to enable stimulation or recordings at the actual interface with the brain have to come first. They need to precede any methods or technologies intended to modify or make use of data and information such technologies provide. Beyond the actual interface itself, mechanical and operational stability and long term reliability of the devices is critical in order to ensure accurate recordings or stimulation. For example, if the electrodes move or there is excessive reactive gliosis it will severely affect the efficacy and accuracy of the devices, rendering any control or adaptation by machine learning algorithms irrelevant. These reflect fundamental engineering challenges that have attracted significant amounts of work. And while significant progress has been made, it very much remains highly active areas of research. We do not discuss these issues further in this paper (see for example Lega et al., 2011; Gilja et al., 2011; Lu et al., 2012).

Beyond these well-known issues surrounding the fabrication and functionality of BMI devices, there are open problems that have received comparatively less attention. Of particular relevance are questions surrounding how data from these devices can be accessed and used, which are of importance to any discussion about integrating machine learning as part of the overall system. At the nanoscale, the density of recording or stimulation can be so large that the telemetry problem of how do you keep track of all those signals becomes an issue. In other words, how do you keep track of where and when signals are coming from (in the case of recordings) or going to (in the case of stimulation). With high density recordings, e.g., many thousands of signals, it becomes physically impossible to follow the standard micro-scale strategy of having individual leads “read out” signals. Most of the nanotechnologies currently being developed for recording at such extreme densities are being engineered as individual standalone nanoscale devices that can then be deployed in large numbers. But even if each individual device is indeed able to faithfully record local signals, how does one extract that information globally across the entire population of sensors and how does one make sense of the resultant data? In the case of applications that necessitate spatial “corticotopic” information, this question is critical. We do not yet have a clear answer, but the impact of the problem cannot be overstated. Whatever the solutions end up being, they will almost certainly necessitate a combination of developments in nanotechnology, algorithms, and data analyses methods. The analogous problem with neural stimulation at nanoscales is how do you selectively target, i.e., turn on and off, nanoscale electrodes in controlled and coordinated spatial and temporal combinations according to defined optimized protocols to produce the most efficient clinically meaningful stimulation paradigms? As discussed above, these would likely differ from patient to patient and evolve over time in the same patient. Being able to accommodate such changes is at the core of the learning and adaptation machine learning methods applied to BMI and neural prosthesis could provide. The design of BMI devices from a materials and engineering standpoint should be aligned with the implementation and integration of machine learning intended to be deployed as part of the overall system.

Other important considerations are broader topics and go beyond just nanoengineered BMI integration with AI, but no less relevant or important. For example, we do not completely understand the neurophysiology, neural code, and intent of neural signals in the context of information processing. This makes it difficult or not yet possible to develop meaningful machine learning algorithms for controlling BMI. So even if the neural stimulation or neural recording interface technologies were perfected, and even if we could develop efficient and accurate machine learning for closed loop feedback control, it still is not clear what we should be optimizing for. We just do not understand how the brain works well enough to do this. With existing neural prosthesis technologies in particular, it is the brain that adapts to the engineered technology, and not the other way around. Other open problems reflect open engineering challenges, beyond the neuroscience. For example, how can machine learning and AI be efficiently implemented on board the device itself given limited form factors and local computational resources? If access to more significant computational resources on the cloud are required, the usual questions of insuring appropriate bandwidth access becomes important, in particular if such reliance was needed under clinically sensitive situations. Is edge computing a possible emerging alternative?

Finally, it is important to acknowledge and consider ethical challenges that arise from the development and use of these technologies. Neurotechnologies and AI on their own each have important ethical considerations. And in at least one recent commentary the ethical considerations of neuroscience, neurotechnologies, and AI were simultaneously discussed (Yuste et al., 2017). Those authors identified four principles that these technologies must adhere to and respect for each individual: privacy, identity, agency and equality. This needs to be an on-going and evolving conversation that tracks with the progress of the technology. The potential risks are too high to ignore or defer.

## Concluding Comments

The integration of machine learning and AI with nanoengineered brain machine and brain computer interfaces offers the potential for significant advances in neurotechnology. BMI’s that have the ability to learn and adapt from the environment and situational demands of external requirements offer tremendous possibilities to radically change the treatment and quality of life of patients. It also offers opportunities for non-invasive interactions and collaborations between humans and machines that at the moment are still in the realm of science fiction. It is conceivable that we are approaching an era of personalized individual experiences that will impact both clinical and non-clinical applications. Of course, as with any truly disruptive and paradigm changing progress, there remain many technical challenges that must be overcome, many in no way trivial or easy, and serious ethical questions that have to be thoughtfully considered and navigated. But it is hard not to be excited about the prospects, what it could mean for how we interact with and use technology and computers for everyone, and the life changing effects it could have on the quality of life and well-being of patients who stand to benefit the most.

We end with one last parting consideration. We have argued the position that the machine learning and AI algorithms that will be required to arrive at “smart” nanoengineered brain machine interface systems may include the use of existing state of the art algorithms, but also possibly new neural derived algorithms and machine learning architectures that more directly model computational and systems neuroscience. What we have not argued for, and what is in no way obvious, is a need for artificial general intelligence (AGI) as necessary to achieve this. Advanced applications such as smart adaptive BMI will almost certainly benefit from advanced algorithms that depend on new mathematical models and theory grounded in empirical neurobiological data. But such algorithms in isolation and out of context do not constitute AGI (although they could conceivably contribute to it). These algorithms need to be able to execute very sophisticated data analyses, pattern recognition, learning, and decision making, but only within the context and embodiment of the neurotechnologies they are supporting. The concept of a self-aware or conscious machine is not required, and should not be confused with the technical considerations that actually are needed, i.e., the discussion in this paper. This distinction is important, because the serious societal and ethical concerns and on-going conversations surrounding AGI are very different than the societal and ethical questions that we need to discuss involving neurotechnologies.

## Author Contributions

The author confirms being the sole contributor of this work and has approved it for publication.

## Conflict of Interest Statement

The author declares that the research was conducted in the absence of any commercial or financial relationships that could be construed as a potential conflict of interest.

## Footnotes

1 http://www.braininitiative.org/

2 https://www.humanbrainproject.eu/en/
